# Preamble to the 2015 SITC immunotherapy biomarkers taskforce

**DOI:** 10.1186/s40425-015-0052-6

**Published:** 2015-03-24

**Authors:** Lisa H Butterfield, Mary L Disis, Bernard A Fox, Samir N Khleif, Francesco M Marincola

**Affiliations:** University of Pittsburgh, Pittsburgh, PA USA; University of Washington, Seattle, WA USA; Oregon Health and Science University, Portland, OR USA; GRU Cancer Center, Georgia Regents University, Augusta, GA USA; Sidra Medical and Research Center, Doha, Qatar; University of Pittsburgh, Hillman Cancer Center, 5117 Centre Avenue, Suite 1.27, Pittsburgh, PA 15213 USA

**Keywords:** Immunotherapy, Biomarkers, Taskforce, Immune monitoring, Clinical trial

## Abstract

The Society for Immunotherapy of Cancer (SITC) has regularly hosted workshops and working groups focused on immunologic monitoring and immune biomarkers. Due to advances in cancer immunotherapy, including positive results from clinical trials testing new agents and combinations, emerging new technologies for measuring aspects of immunity, and novel candidate biomarkers from early phase trials, the SITC Immune Biomarkers Taskforce has reconvened to review the state of the art, identify current hurdles to further success and to make recommendations to the field. Topics being addressed by individual working groups include: (1) validation of candidate biomarkers, (2) identification of the most promising technologies, (3) testing of high throughput immune signatures and (4) investigation of the pre-treatment tumor microenvironment. Resultant recommendations will be published in JITC.

## Background

In the past five years, the field of cancer immunotherapy has been revolutionized by new clinical successes. These improved outcomes for cancer patients are due to several years of development of new drugs, preclinical studies and large scale clinical trials. New immunotherapeutics have been approved by the FDA, including antibodies that block immune checkpoint molecules CTLA-4 and PD-1. Furthermore, very impressive durable responses have been obtained after adoptive transfer of several types of cellular populations, including genetically engineered lymphocytes.

These clinical advances and newly approved therapeutics are moving cancer immunotherapy from a niche approach in limited centers, primarily for melanoma and renal cancer, to a broadly applicable and effective treatment for many common and deadly cancers. Despite the success, the current drugs and early data from their various combinations do not result in durable responses for every patient, and they are not without toxicity. Therefore, a critical roadblock in cancer therapy remains the identification of patients likely to respond to a given immunotherapy who could be pre-selected. In addition, parameters need to be sought to follow patients during therapy to identify those that are benefitting before a clinical response is evident. This is because, contrary to other treatments, evaluable clinical responses to immunotherapy often lag in time due to secondary biologic activity of tumors due to infiltration by inflammatory cells [1].

The Society for Immunotherapy of Cancer (SITC) has been investigating predictive and prognostic biomarkers of response in cancer immunotherapy for many years. The Society has focused on immune biomarkers in several dedicated workshops [2-4]. Between 2008–2011, the SITC Taskforce on Immunotherapy Biomarkers developed a series of recommendations and resources for the field [5-7]. Since then, the field has progressed further. New therapeutics are now in common use and technology has evolved significantly. In addition, a greater understanding of the tumor microenvironment now exists, and characterization of tumor infiltrating cell populations and immune regulatory molecules expressed by both the tumor and TIL is more commonly performed [8-11].

As a field, we still lack the necessary biomarkers that identify the patients who are most likely to respond to a given approach and who will have minimal or acceptable toxicities, nor do we have defined, standardized and validated biomarkers to identify the patients who are responding to a treatment before clinically evident tumor shrinkage is detected. There are, however, many candidate assays and molecules which seem informative in a given trial. These include standardized measures for antigen-specific T cell activation, frequencies of suppressive Treg and MDSC, circulating cytokines and growth factors, antibody titers, and tumor immune cell infiltrate characterization. However, most studies only test one or two readouts, tumor samples are often not available, blood volumes can be limited, and methods for performing assays and interpreting the results vary broadly. Furthermore, none of these tests have definitively proven to date to correlate with clinical outcome across trials.

To address these issues, the SITC Immune Biomarkers Task Force has reconvened under the continuing leadership of the Steering Committee: Lisa H. Butterfield, Mary L. Disis, Bernard A. Fox, Samir N. Khleif and Francesco M. Marincola. In its previous iteration, the Taskforce had two working groups which individually addressed two general areas. Working Group (WG)1, led by Drs. Butterfield and Disis, focused on issues of standardization and validation of immune assays. WG2, led by Drs. Marincola and Peter P. Lee, focused on novel technologies and high throughput approaches.

The recommendations of the Taskforce, published in 2011 [6] could be summarized as follows:

“Although specific immune parameters and assays are not yet validated, we recommend following standardized (accurate, precise, and reproducible) protocols and use of functional assays for the primary immunologic readouts of a trial; consideration of central laboratories for immune monitoring of large, multi-institutional trials; and standardized testing of several phenotypic and functional potential potency assays specific to any cellular product. When reporting results, the full QA (quality assessment)/QC (quality control) should be conducted and selected examples of truly representative raw data and assay performance characteristics should be included. Finally, to promote broader analysis of multiple aspects of immunity, and gather data on variability, we recommend that in addition to cells and serum, RNA and DNA samples be banked (under standardized conditions) for later testing. We also recommend that sufficient blood be drawn to allow for planned testing of the primary hypothesis being addressed in the trial, and that additional baseline and post-treatment blood is banked for testing novel hypotheses (or generating new hypotheses) that arise in the field”.

### Goals of the task force in 2015

For 2015, the SITC Immune Biomarkers Taskforce is divided into four different WG, reflecting the growth and development of the field of cancer immunotherapy and the increased understanding of the reach of the immune system into other areas, like tumor-specific mutations arising during carcinogenesis, the microbiome and the impact of immunity on responses to tumor signaling pathway modulation with small molecules and antibodies.

WG1, led by Drs. Magdalena Thurin and Giuseppe Masucci, is focusing on immunologic monitoring assay standardization and validation. This group includes experts from the US National Cancer Institute (NCI), academia, the US FDA, biotech and pharma. They are developing roadmaps for standardization and validation of specific assays that are currently seen as the most promising, particularly with companion diagnostics as the goal. They are also examining proficiency panels of assays, and “integral” and “integrated” (versus exploratory) biomarkers in clinical trials, and statistical designs for biomarker validation.

WG2, led by Dr. Jianda Yuan, is focused on new developments in biomarker assays and new technologies. This group also has broad international expertise from academia and industry, including specialists in specific technologies like epigenetics, TCR sequencing and flow cytometry. They are focusing their work on identifying the most promising new technologies that can be applied to clinical trials of immunotherapeutic agents, in particular checkpoint blockade. They are also making recommendations regarding sample handling and data analysis.

WG3, led by Dr. David Stroncek, is focusing on the assessment of immune regulation and modulation systematically. This group has expertise from the NCI, as well as representatives from international academic and pharmaceutical companies. They are reviewing the state of the art of high throughput technologies and recent studies identifying signatures of immune function, immune response and clinical outcomes in cancer patients. They are also examining roles for proteomics, metabolomics and the microbiome in understanding immune responses in patients.

WG4, led by Dr. Sacha Gnjatic, is focusing on prediction of clinical outcome based on baseline measures, particularly those in the tumor microenvironment. This WG also includes participants with a broad range of expertise in both blood and tumor immune assessment, including faculty from a number of international academic sites as well as biotech company and pharma representatives. They are examining blood, genetic, transcriptomic, proteomic and immune infiltrate assessments and signatures to address identification of patients suitable for different treatment approaches.

Each WG is charged with reviewing the state of the art for their area, identifying the current scientific and technical hurdles and making recommendations on how best to move forward. These recommendations will be presented in a white paper from each WG, to appear in the JITC. Another aspect of the Taskforce function is to update the SITC membership and JITC readership with published brief synopses of new immune monitoring technologies, as each WG operates in the coming months.

Lastly, members of the Taskforce have received regulatory approvals to access banked PBMC and serum samples from a recently completed clinical trial. This multi-institution immunotherapy trial for melanoma tested CTLA-4 blockade +/− the addition of GM-CSF in a 2-arm trial that included 245 melanoma patients, which was run with the support of the ECOG-ACRIN national cooperative group. The initial clinical trial report from F.S. Hodi et al. [12] was recently published. The blood samples, which are banked at the Immunology Reference Lab for ECOG-ACRIN at the University of Pittsburgh Cancer Institute, represent an important resource for immune biomarker testing given the clinical outcomes and toxicity differences. Drs. Butterfield and Marincola will lead this effort, testing both standardized cellular immunity and circulating cytokine/chemokine/growth factor assays (Butterfield/Pittsburgh) and high throughput genomic and transcriptomic measures (Marincola/Sidra). This clinical trial project will also benefit from the discussions of each WG. The results will also appear in JITC when complete.

## Conclusion

Together, the SITC Immune Biomarkers Taskforce will provide the field of cancer immunotherapy with continuous status updates on critical immune biomarkers which are on the path to validation as well as a roadmap for other established and standardized biomarkers to become validated. The newest technologies and high throughput approaches, with their strengths and weaknesses and sample handling requirements will be presented. The latest investigations in the tumor microenvironment and the most informative baseline immune measures will also be presented. The Taskforce will not only publish summaries and recommendations but will also host a day long workshop for open discussion with the broader field stake holders, planned for the Spring of 2016. Lastly, we will use this Taskforce as the platform for performing new immune biomarker studies in a large, multi-institution immunotherapy trial in melanoma. These banked patient samples provide a critical opportunity for putting the standardized assays and new and high throughput technologies to the test.

## Abbreviations

SITC: Society for Immunotherapy of Cancer
JITC: Journal for Immunotherapy of Cancer
FDA: Food and Drug Administration
CTLA-4: Cytotoxic T lymphocyte associated protein 4
PD-1: Programmed death-1
WG: Working group
TIL: Tumor infiltrating lymphocytes
QA: Quality assurance
QC: Quality control
NCI: National Cancer Institute
TCR: T cell receptor
GM-CSF: Granulocyte-macrophage colony stimulating factor
ECOG: Eastern Cooperative Oncology Group
ACRIN: American College of Radiology Imaging Network
